# Patterns of engagement in HIV care during pregnancy and breastfeeding: findings from a cohort study in North-Eastern South Africa

**DOI:** 10.1186/s12889-021-11742-4

**Published:** 2021-09-21

**Authors:** David Etoori, Brian Rice, Georges Reniers, Francesc Xavier Gomez-Olive, Jenny Renju, Chodziwadziwa Whiteson Kabudula, Alison Wringe

**Affiliations:** 1 Department of Population Health, London School of Hygiene and Tropical Medicine, London, UK; 2 MeSH Consortium, Department of Public Health Environments and Society, Faculty of Public Health and Policy, London School of Hygiene and Tropical Medicine, London, UK; 3 MRC/Wits Rural Public Health and Health Transitions Research Unit (Agincourt), School of Public Health, Faculty of Health Sciences, University of Witwatersrand, Johannesburg, South Africa; 4 Kilimanjaro Christian Medical University College, Moshi, Tanzania

**Keywords:** Pregnancy, ART, Loss to follow-up, Mother-to-child transmission, South Africa

## Abstract

**Background:**

Eliminating mother-to-child transmission of HIV (MTCT) in sub-Saharan Africa is hindered by limited understanding of HIV-testing and HIV-care engagement among pregnant and breastfeeding women.

**Methods:**

We investigated HIV-testing and HIV-care engagement during pregnancy and breastfeeding from 2014 to 2018 in the Agincourt Health and Demographic Surveillance System (HDSS). We linked HIV patient clinic records to HDSS pregnancy data. We modelled time to a first recorded HIV-diagnosis following conception, and time to antiretroviral therapy (ART) initiation following diagnosis using Kaplan-Meier methods.

We performed sequence and cluster analyses for all pregnancies linked to HIV-related clinic data to categorise MTCT risk period engagement patterns and identified factors associated with different engagement patterns using logistic regression.

We determined factors associated with ART resumption for women who were lost to follow-up (LTFU) using Cox regression.

**Results:**

Since 2014, 15% of 10,735 pregnancies were recorded as occurring to previously (51%) or newly (49%) HIV-diagnosed women. New diagnoses increased until 2016 and then declined. We identified four MTCT risk period engagement patterns (i) early ART/stable care (51.9%), (ii) early ART/unstable care (34.1%), (iii) late ART initiators (7.6%), and (iv) postnatal seroconversion/early, stable ART (6.4%). Year of delivery, mother’s age, marital status, and baseline CD4 were associated with these patterns. A new pregnancy increased the likelihood of treatment resumption following LTFU.

**Conclusion:**

Almost half of all pregnant women did not have optimal ART coverage during the MTCT risk period. Programmes need to focus on improving retention, and leveraging new pregnancies to re-engage HIV-positive women on ART.

**Supplementary Information:**

The online version contains supplementary material available at 10.1186/s12889-021-11742-4.

## Background

In sub-Saharan Africa (SSA) mother-to-child transmission (MTCT) rates ranged from 1.9% in Botswana to 31.9% in Somalia in 2019 [1]. The risk of MTCT starts at conception and continues until breastfeeding cessation, which ranges from 18 to 24 months in SSA countries [2–4]. Without intervention, MTCT risk is estimated at 5–10% during pregnancy, 10–15% during delivery, and 5–20% during breastfeeding, with the cumulative risk being 30–45% for infants who are breastfed for 18–24 months [5]. The adoption of Option B+ by most treatment programmes in SSA, including South Africa in 2015, has led to large reductions in MTCT rates [6, 7]. Option B+ has dramatically increased the number of pregnant women initiating ART, however retention is challenging, particularly in the postnatal period [8]. Reasons for disengagement include still feeling healthy, transition from PMTCT to adult ART services, stigma, and denial of HIV status [9–12]. In 2019, the United Nations Children’s Emergency Fund (UNICEF) released their roadmap for the elimination of mother-to-child transmission of HIV (EMTCT) [13], which identified missed opportunities for EMTCT including failure to prevent seroconversion during pregnancy or breastfeeding, mothers living with HIV (MLHIV) who do not receive antiretroviral therapy (ART), MLHIV who previously started ART and stopped, and MLHIV who started ART late in their pregnancy.

An increase in the proportion of people initiating ART shortly after being diagnosed with HIV, as per World Health Organisation (WHO) guidance [14], has in turn led to an increased spectrum of engagement in care patterns, with treatment interruption, reengagement in care and clinic transfers becoming increasingly prevalent [15–18]. Previous studies have used cohort and cross-sectional data to focus on the occurrence of specific events along the care cascade including linkage to care, ART initiation, and ART discontinuation [7, 19, 20]. However, these snapshots do not always capture the complexity of individual care trajectories. Monitoring these trajectories and understanding the factors that underlie patterns of disengagement and reengagement in care is becoming increasingly important to inform interventions to accelerate progress towards the second and third targets of the UNAIDS 95–95-95 goals and to reduce MTCT [21].

To inform interventions to address missed opportunities for EMTCT, we investigated HIV testing and engagement in care for pregnant and breastfeeding women in north-eastern South Africa. Using linked demographic surveillance and health facility data, we report on HIV testing events, identify periods of ART engagement and discontinuation during the pregnancy and postpartum periods for women living with HIV (WLHIV), and investigate factors associated with ART resumption during the MTCT risk period.

## Methods

### Setting and data sources

The Agincourt Health and Demographic Surveillance System (HDSS) is approximately 525 km^2^ with an estimated population of 120,000 residents living in 20,000 households within 31 villages [22]. The HDSS is located in Mpumalanga province, South Africa with HIV prevalence estimated at 14.1% across all ages [22–24]. In 2013, the total fertility rate was estimated as 2.4 children per woman [25]. Data sources and data used are summarised in Additional file 1.

#### Demographic surveillance

Fertility, mortality and migration data are collected annually from residents, based on a comprehensive household registration system, in operation since 1992 [22, 26]. Fieldworkers visit each household and interview the most knowledgeable adult available to obtain information on demographic events occurring since the last census [22, 27, 28]. The HDSS also collects verbal autopsy data to ascertain probable causes of death [29, 30]. Wealth quintiles were calculated using a list of household asset indicators collected as part of demographic surveillance. These calculations have been described in more detail elsewhere [30].

#### Point-of-contact interactive record linkage (PIRL)

Since 2014, HIV patient visits to ART health facilities in the area have been logged by fieldworkers and linked to HDSS resident records using Point-of-Contact Interactive Record Linkage (PIRL), described in detail elsewhere [31, 32]. In brief, a fieldworker conducts a short uptake interview with patients in the health facility waiting area. Consenting patients are asked for personal identifiers used to search the HDSS database using a probabilistic algorithm. Matches are confirmed in interaction with the patients, and names of household members are used as a key attribute to adjudicate between possible matches. Linkage data extraction for these analyses occurred on July 11, 2018.

#### Record review and patient tracing study

Lost to follow-up (LTFU) data are drawn from a study described in detail elsewhere [33, 34]. Briefly, on August 15, 2017, all patients within the PIRL database who were considered LTFU (more than 90 days late for a scheduled health facility visit) were recruited into a cohort and followed up to ascertain whether they were still alive and on treatment.

### Data preparation and statistical analysis

All pregnancies occurring from 2014 onwards, recorded in the demographic surveillance database and ending in a live birth were eligible for this analysis. Women could contribute more than one pregnancy. Pregnancy data extraction for these analyses occurred on January 31, 2020, with the last recorded delivery occurring on November 11, 2018.

The Agincourt HDSS does not routinely conduct HIV serological surveys among its population. However, the availability of PIRL data presented a unique opportunity to update prevalence estimates [35], with linkage to an HIV-treatment health facility record presumed to indicate an HIV diagnosis. We identified HIV-treatment health facility records linked to each pregnancy through the PIRL database to determine dates of HIV diagnosis, ART initiation, and all HIV-associated health facility visits. Where no ART initiation date was reported, the first ART-related health facility visit date was used as the ART initiation date. Where the HIV diagnosis date was missing, it was assumed to have occurred the day before ART initiation (the most common occurrence for individuals with both HIV diagnosis and ART initiation dates available in our data). Conception for each pregnancy was estimated to have occurred 280 days prior to the delivery date. Demographic and health surveys data suggests that 10% of babies were still breastfed at 23 months in South Africa in 2016 [36], therefore, breastfeeding cessation was set at 24 months (730 days) following the delivery date to give the longest possible period of transmission risk.

#### Seroconversions and ART initiation

Any pregnancy for which an HIV diagnosis date occurred before the conception date was excluded from seroconversion analyses. Time to a first recorded HIV-positive result was calculated with the estimated conception date as the origin. We also calculated time to ART initiation with the date of the first recorded HIV-positive result as the origin. These time-to-event data were modelled using Kaplan-Meier methods.

#### Sequence states and outcomes

All pregnancies for which a first HIV-positive result was recorded before breastfeeding cessation were considered for this analysis. Each pregnancy was assigned a 1010-day sequence corresponding to 280 days of pregnancy and 730 days of breastfeeding. There were nine possible sequence states (Table 1).

**Table 1:** Definitions of all the possible sequence states

State	Definition
Unknown HIV	No health facility records of an HIV diagnosis, or ART initiation before the pregnancy or breastfeeding period.
HIV+ no ART	Following HIV diagnosis, ART not initiated, or ART initiated but without any follow-up visits immediately following ART initiation.
On ART	At least one health facility visit following the ART initiation visit. Women remained on ART up to their next scheduled visit.
Late	Between 7 and 90 days late for a scheduled health facility visit following ART initiation.
LTFU	More than 90 days late for a scheduled health facility visit following ART initiation.
Deceased	From the date of death extracted from HDSS records.
Transferred	A record of treatment collection from a health facility within the Agincourt HDSS other than where ART initiation occurred.
Reengaged	Restarted treatment at the same health facility where ART initiation occurred following LTFU.
Migration	Out migrated from the HDSS with no evidence of health facility visits following the migration date.

Using a state sequence-analysis approach, and utilising Optimal Matching distances, we identified distinct clusters of sequences with similar patterns of engagement in care during the MTCT risk period. Using logistic regression, we determined factors associated with belonging to a given cluster, after multiple imputation to account for missingness in the explanatory variables.

#### Factors associated with ART resumption

Using the record review and tracing study data, we restricted these analyses to women of reproductive age who were considered LTFU. Resumption of ART was defined as either reengagement or transfer, following loss to follow-up. Follow-up time began on the date of each patient’s last recorded clinic visit. Follow-up time was split into monthly intervals to account for pregnancy which was included as a time-varying covariate. We linked pregnancy data from the HDSS to identify new pregnancies, defined as a pregnancy occurring after LTFU that was not associated with previous HIV-treatment episodes. Women were considered pregnant if a monthly interval fell within the period of a new pregnancy identified through HDSS data. All other covariates remained constant in each time interval. A Cox regression model was used to determine the factors associated with ART resumption, with all other outcomes considered to be right-censored. Bi-variate analyses were conducted with a priori selected variables for which there was a plausible association to ART resumption. All variables with *p* < 0.1 were included in the multivariable Cox regression model. A parsimonious model was achieved using Wald tests. All models accounted for clustering at the clinic level and utilised robust standard errors.

Sequence analyses were conducted using the *TraMineR* R-package [37]. All other analyses were conducted using Stata 16 [38].

## Results

### Population characteristics

Between January 2014 and November 2018, 10,102 women had 10,831 pregnancies recorded in the HDSS, among which 96 (0.9%) ended in a still birth or abortion. The remaining 10,735 live births were recorded for 10,035 women (Table 2). Median age at delivery was 26 years (IQR: 21, 32).

**Table 2:** Sociodemographic characteristics of all pregnancies recorded in the Agincourt HDSS since 2014

	Not linked to HIV-related health record (%)	First recorded HIV+ test				Total (%)
		Before conception (%)	During pregnancy (%)	During breastfeeding period (%)	After breastfeeding period (%)	
Pregnancies	9107 (84.8)	823 (7.7)	585 (5.4)	127 (1.2)	93 (0.9)	10,735
Delivery year						
2014	2248 (83.9)	154 (5.8)	156 (5.8)	53 (2.0)	67 (2.5)	2673
2015	2046 (84.4)	167 (6.9)	145 (6.0)	39 (1.6)	26 (1.1)	2423
2016	1888 (84.6)	182 (8.2)	139 (6.2)	23 (1.0)	0 (0)	2232
2017	1749 (85.7)	185 (9.1)	94 (4.6)	12 (0.6)	0 (0)	2040
2018	1176 (86.3)	135 (9.9)	51 (3.7)	0 (0)	0 (0)	1362
Age group						
10–14	32 (100.0)	0 (0)	0 (0)	0 (0)	0 (0)	32
15–19	1663 (95.0)	20 (1.1)	33 (1.9)	18 (1.0)	17 (1.0)	1751
20–24	2517 (89.8)	96 (3.4)	132 (4.7)	32 (1.1)	27 (1.0)	2804
25–29	2244 (84.0)	185 (6.9)	184 (6.9)	34 (1.3)	24 (0.9)	2671
30–34	1517 (78.0)	244 (12.5)	141 (7.2)	26 (1.3)	17 (0.9)	1945
35–39	804 (73.4)	202 (18.4)	72 (6.6)	10 (0.9)	7 (0.6)	1095
40+	330 (75.5)	76 (17.4)	23 (5.3)	7 (1.6)	1 (0.2)	437
Gravida						
1	6462 (88.9)	315 (4.3)	345 (4.7)	83 (1.1)	61 (0.8)	7266
2	1961 (78.3)	318 (12.7)	171 (6.8)	29 (1.2)	26 (1.0)	2505
3	474 (71.2)	135 (20.3)	47 (7.1)	8 (1.2)	2 (0.3)	666
4	133 (66.5)	41 (20.5)	17 (8.5)	6 (3.0)	3 (1.5)	200
5	45 (71.4)	11 (17.5)	5 (7.9)	1 (1.6)	1 (1.6)	63
6	24 (88.9)	3 (11.1)	0 (0)	0 (0)	0 (0)	27
7	6 (100.0)	0 (0)	0 (0)	0 (0)	0 (0)	6
8	2 (100.0)	0 (0)	0 (0)	0 (0)	0 (0)	2
Planned pregnancy						
No	4170 (85.2)	357 (7.3)	258 (5.3)	65 (1.3)	44 (0.9)	4894
Yes	3998 (83.7)	398 (8.3)	282 (5.9)	53 (1.1)	46 (1.0)	4777
Missing	939 (88.3)	68 (6.4)	45 (4.2)	9 (0.8)	3 (0.3)	1064
Education						
No formal education	97 (76.4)	15 (11.8)	9 (7.1)	4 (3.1)	2 (1.6)	127
Primary	1289 (79.2)	172 (10.6)	118 (7.2)	28 (1.7)	21 (1.3)	1628
Secondary	6303 (85.8)	524 (7.1)	385 (5.2)	77 (1.0)	56 (0.8)	7345
Tertiary	599 (92.7)	21 (3.3)	22 (3.4)	3 (0.5)	1 (0.2)	646
Missing	819 (82.8)	91 (9.2)	51 (5.2)	15 (1.5)	13 (1.3)	989
Marital status						
Single	5902 (85.4)	492 (7.1)	369 (5.3)	89 (1.3)	58 (0.8)	6910
Married	1312 (88.8)	91 (6.2)	56 (3.8)	11 (0.7)	8 (0.5)	1478
Informal union	1390 (84.2)	129 (7.8)	98 (5.9)	16 (1.0)	18 (1.1)	1651
Separated	359 (77.9)	57 (12.4)	33 (7.2)	4 (0.9)	8 (1.7)	461
Divorced	78 (60.9)	28 (21.9)	17 (13.3)	4 (3.1)	1 (0.8)	128
Widowed	66 (61.7)	26 (24.3)	12 (11.2)	3 (2.8)	0 (0)	107
Wealth quintiles						
1	1369 (82.2)	156 (9.4)	103 (6.2)	25 (1.5)	12 (0.7)	1665
2	1607 (82.4)	167 (8.6)	130 (6.7)	26 (1.3)	21 (1.1)	1951
3	1715 (85.0)	150 (7.4)	105 (5.2)	24 (1.2)	24 (1.2)	2018
4	1895 (85.2)	167 (7.5)	117 (5.3)	25 (1.1)	21 (0.9)	2225
5	1967 (88.2)	135 (6.1)	98 (4.4)	20 (0.9)	10 (0.4)	2230
Missing	554 (85.8)	48 (7.4)	32 (5.0)	7 (1.1)	5 (0.8)	646

### Seroconversions and ART initiation

Of the 10,735 pregnancies ending in live births, 1628 (15.2%) were linked to an HIV-related health facility record. Of these, 823 (50.6%) had a first HIV-positive test recorded before the estimated conception date and were excluded from Kaplan-Meier (KM) models, 585 (35.9%) had a first test recorded during the pregnancy, 127 (7.8%) had a first test recorded during the breastfeeding period, and 93 (5.7%) had a first test occurring after breastfeeding cessation.

**Fig. 1:**
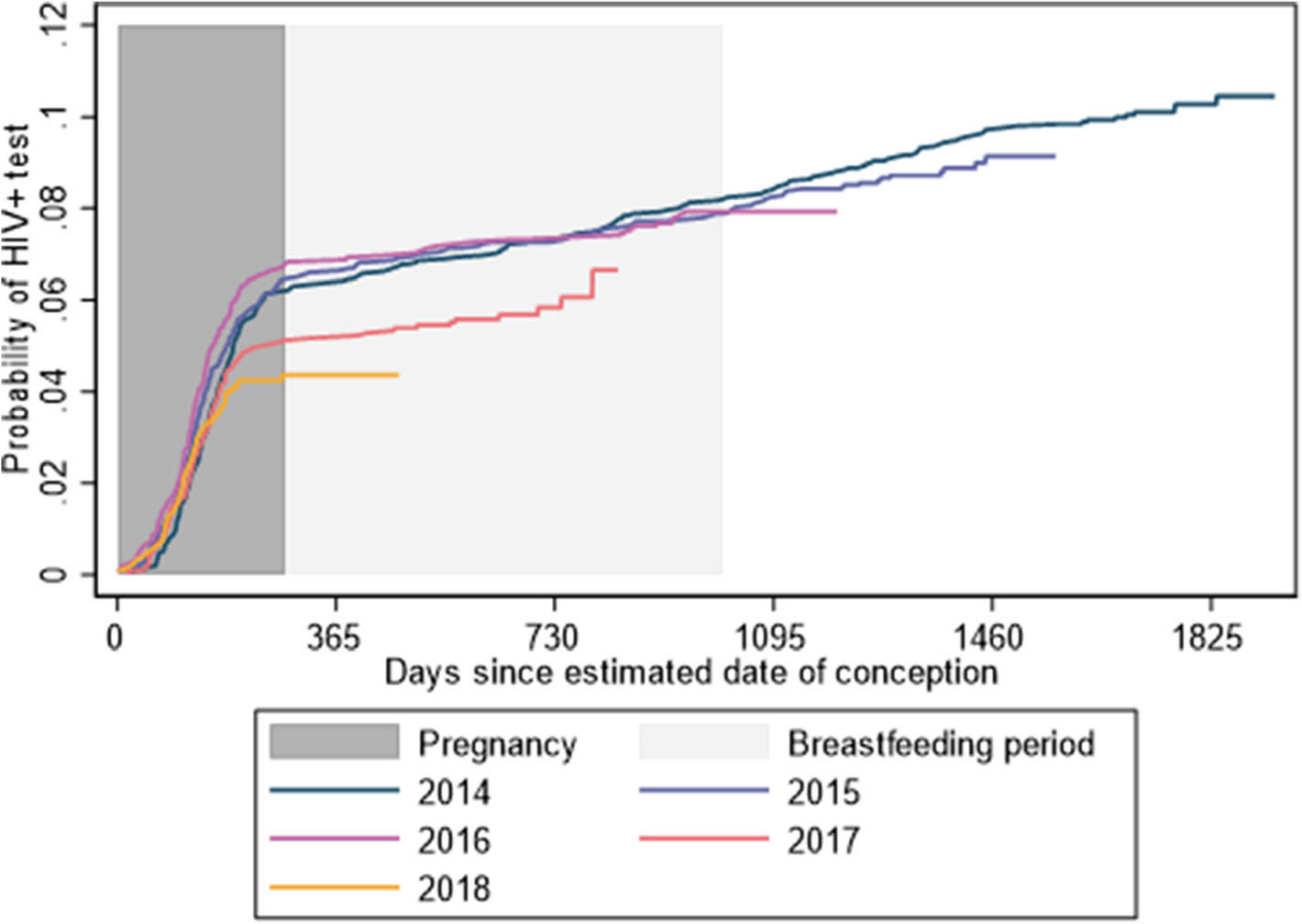
Kaplan-Meier curves showing the probability of an HIV-positive test during the vertical transmission risk period stratified by the year of delivery. This figure shows the cumulatative probability of having an HIV positive test linked to a pregnant or postpartum woman given there was no previous record or evidence of HIV diagnosis or clinic attendance for HIV care. This cumulative probability is stratified by delivery year showing a reduction in the cumulative probability in 2017 and 2018

For women contributing more than one pregnancy to the analysis, previous pregnancies contributed analysis time up to the estimated conception date of the next pregnancy. The cumulative probability of having a positive HIV test by the end of the most recent pregnancy was 5.95, and 7.47% by the end of breastfeeding. The probability of having an HIV positive test varied by year of delivery (with a 1.5% reduction between 2016 and 2017 (*p* = 0.0201)) (Fig. 1), was higher for older women peaking in the 30–34 age group, for women in lower wealth quintiles, for divorced or widowed women, and for less educated women (all *p* < 0.001).

The cumulative probability of ART initiation following a recorded HIV-positive test during pregnancy was 50.59% after 43 days, 75.04% after 202 days, 90.15% after 547 days, and 100% after 1687 days. This probability increased by year of delivery with a significant reduction in initiation time from 2016 (< 0.001) (Additional file 2).

### Engagement sequences and clusters

We identified distinct patterns of clinic attendance connected to the pregnancy and breastfeeding period (Additional file 3), with 1477 women contributing 1628 pregnancies which were treated as independent for the purpose of this analysis. Of 1628 pregnant women, 93 were excluded from these analyses because the first recorded HIV positive test occurred after the breastfeeding period.

**Fig. 2:**
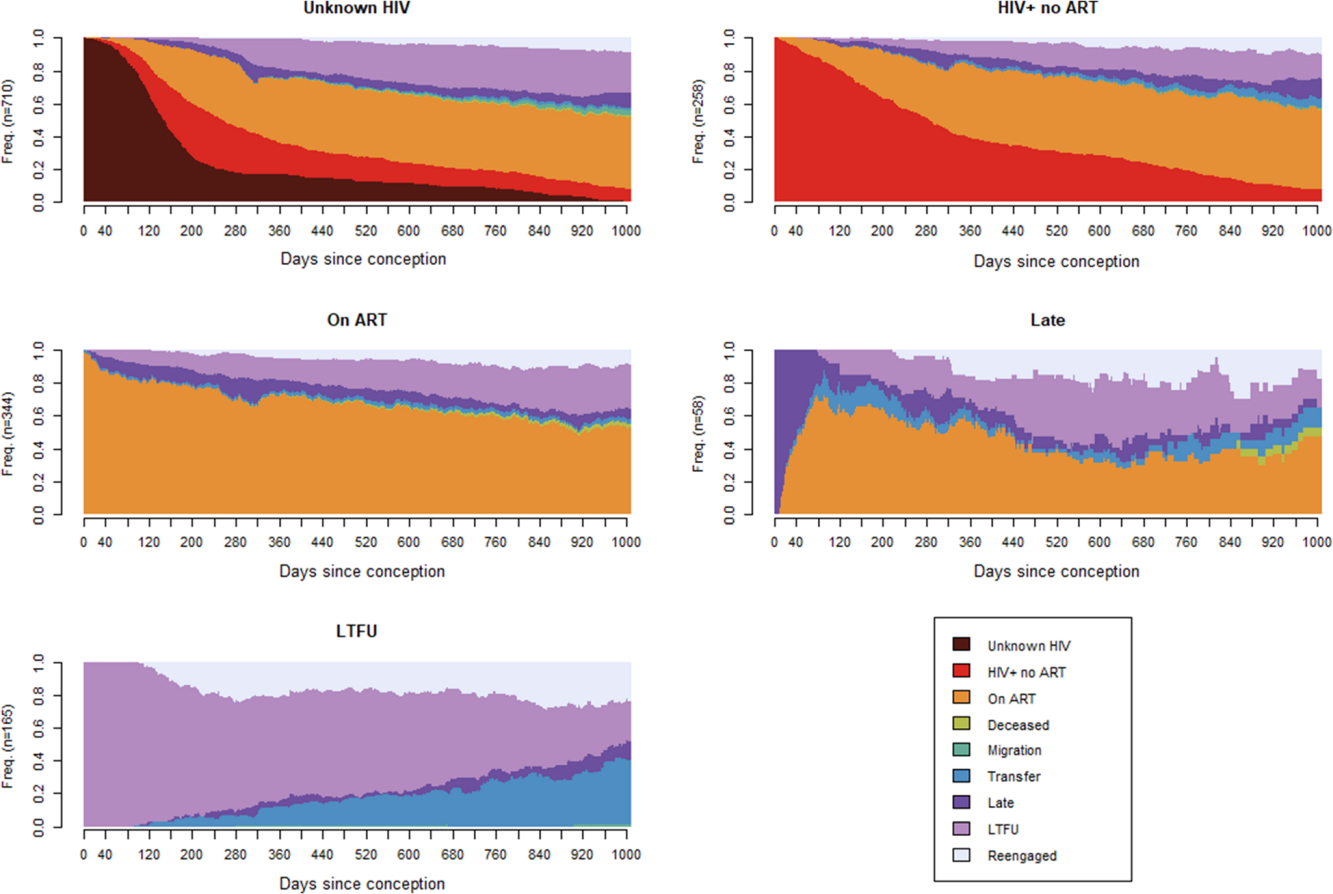
Chronograms of engagement in care during the vertical risk transmission period by status at the beginning of the pregnancy

Of the remaining 1535 pregnant women, 710 (46.3%) had an unknown HIV status, 258 (16.8%) were HIV-positive but not on ART, 344 (22.4%) were on ART, 58 (3.8%) were late for their most recent appointment, and 165 (10.7%) were LTFU from HIV care at the beginning of their pregnancy. There was a yearly increase in the proportion of women already on ART at the beginning of their pregnancy starting at 0% in 2014 and increasing to 39.25% by 2018 (*p* < 0.001). Of 258 WLHIV but not on ART when they became pregnant, 133 (51.5%) had not initiated ART at delivery. Of 165 WLHIV who were LTFU when they became pregnant, 109 (66.1%) were still LTFU at delivery, however, 148 (89.7%) resumed ART by the end of the breastfeeding period (Fig. 2).

The highest resumption happened in the 6 months leading up to delivery (33 (20.0%) second trimester, 28 (17%) third trimester), was its lowest in the 3 months after delivery (17 (10.3%) pregnant women) and increased during the breastfeeding period before levelling off (Additional file 4).

Cluster analysis identified four distinct patterns of engagement in care in the MTCT risk period: (i) women who initiated ART early and were stable in care (*n* = 796, 51.9%), (ii) women who initiated ART early but were not stable in care (characterised by frequent ART stoppages or late appointment attendance) (*n* = 524, 34.1%), (iii) women who were late ART initiators (*n* = 117, 7.6%), and (iv) postnatal seroconversion with early, stable ART (*n* = 98, 6.4%). In the logistic regression model, older women and women who delivered more recently were more likely to be in the early stable ART group. Younger women were more likely to seroconvert postnatally. Women who delivered in 2015 and 2016 were least likely to be in the late ART group or to seroconvert postnatally. Married women and women with a baseline CD4 > 500 cells/ μL were more likely to initiate ART late. (Additional file 5).

### Factors associated with ART resumption

Of 767 (75.4%) women recorded as LTFU in the PIRL database, 62 (8.1%) were older than reproductive age (> 51 years) and were excluded from this analysis. Of the remaining 705, 279 (39.6%) initiated ART for PMTCT, 354 (50.2%) had been LTFU for less than a year, 468 (66.4%) had a baseline CD4 > 200 cells/ μL, and 84 (11.9%) had a pregnancy occurring during the follow-up time. The median age was 31 years (IQR: 27, 36) (Table 3).

**Table 3:** Factors associated with resumption of ART following loss to follow-up

	LTFU	HR (95% CI)	*p*-value	aHR (95% CI)	*p*-value
	705				
	N (%)				
Pregnancy during follow-up time					
No	621 (88.1)	Reference	__	Reference	__
Yes	84 (11.9)	2.85 (1.50, 5.43)	0.001	2.79 (1.31, 5.95)	0.008
Age					
18–24	117 (16.6)	0.81 (0.44, 1.50)	0.511		
25–34	379 (53.8)	Reference	__		
35–44	161 (22.8)	1.01 (0.78, 1.31)	0.927		
45–51	48 (6.8)	0.88 (0.62, 1.24)	0.462		
ART reason					
Non-PMTCT	426 (60.4)	Reference	__		
PMTCT	279 (39.6)	0.78 (0.66, 0.92)	0.003		
ART start year					
2014	144 (20.4)	1.18 (0.69, 1.99)	0.545	0.88 (0.48, 1.63)	0.69
2015	288 (40.8)	Reference	__	Reference	__
2016	248 (35.2)	1.36 (1.07, 1.71)	0.011	2.17 (1.28, 3.67)	0.004
2017	25 (3.6)	1.38 (0.72, 2.64)	0.324	3.08 (1.07, 8.84)	0.037
Time on ART					
≤ 3 months	208 (29.5)	Reference	__	Reference	__
3–6 months	150 (21.3)	0.90 (0.61, 1.33)	0.599	1.06 (0.71, 1.58)	0.78
6–12 months	174 (24.7)	1.70 (1.09, 2.65)	0.018	1.98 (1.20, 3.26)	0.008
12–24 months	139 (19.7)	3.29 (1.44, 7.51)	0.005	4.43 (1.66, 11.84)	0.003
> 24 months	34 (4.8)	5.73 (3.60, 9.10)	< 0.001	7.90 (3.56, 17.51)	< 0.001
Baseline CD4					
< 100	105 (15.4)	0.67 (0.34, 1.32)	0.244	0.76 (0.37, 1.59)	0.474
100–199	107 (15.7)	0.82 (0.62, 1.08)	0.152	0.87 (0.62, 1.22)	0.416
200–349	183 (26.9)	Reference	__	Reference	__
350–499	159 (23.4)	0.90 (0.68, 1.19)	0.461	1.17 (0.82, 1.67)	0.395
500+	126 (18.5)	1.48 (1.01, 2.17)	0.044	1.76 (1.40, 2.20)	< 0.001
Baseline WHO stage					
I	554 (79.6)	Reference	__		
II	75 (10.8)	1.27 (0.93, 1.73)	0.136		
III	62 (8.9)	1.29 (0.60, 2.74)	0.512		
IV	5 (0.7)	0.91 (0.24, 3.43)	0.885		
Refill schedule					
1 month	469 (66.5)	Reference	__	Reference	__
2 months	157 (22.3)	0.93 (0.56, 1.56)	0.791	0.86 (0.47, 1.60)	0.646
3 months	60 (8.5)	1.29 (0.94, 1.78)	0.112	0.88 (0.54, 1.44)	0.604
> 3 months	19 (2.7)	6.42 (4.24, 9.73)	< 0.001	2.79 (2.11, 3.69)	< 0.001
Health Facility					
Agincourt	195 (27.7)	Reference	__	Reference	__
Belfast	135 (19.2)	0.36 (0.34, 0.39)	< 0.001	0.48 (0.41, 0.57)	< 0.001
Cunningmore	44 (6.2)	0.37 (0.35, 0.40)	< 0.001	0.65 (0.53, 0.80)	0.001
Justicia	75 (10.6)	0.32 (0.28, 0.36)	< 0.001	0.53 (0.45, 0.61)	< 0.001
Kildare	75 (10.6)	0.84 (0.83, 0.86)	< 0.001	0.96 (0.73, 1.27)	0.804
Lillydale	110 (15.6)	0.65 (0.62, 0.68)	< 0.001	1.01 (0.88, 1.16)	0.854
Thulamahashe	21 (3.0)	0.64 (0.63, 0.65)	< 0.001	0.67 (0.56, 0.81)	< 0.001
Xanthia	50 (7.1)	0.60 (0.59, 0.61)	< 0.001	0.59 (0.45, 0.76)	< 0.001
Time since last appointment					
≤ 1 year	354 (50.2)	Reference	__	Reference	__
1–2 years	269 (38.2)	0.36 (0.28, 0.46)	< 0.001	0.63 (0.49, 0.80)	< 0.001
> 2 years	82 (11.6)	0.10 (0.06, 0.17)	< 0.001	0.33 (0.16, 0.65)	0.001

Of these 705 women, 247 (35.0%) were found to have resumed ART (147 (59.5%) reengaged and 100 (40.5%) transferred). Women were out of care a median of 312 days (IQR: 182, 541) before resuming ART (Reengaged: 333 (170, 525), Transferred: 297.5 (185, 563), *p* = 0.6482). In the multivariable Cox model, a new pregnancy was associated with ART resumption (aHR: 2.79, 95% C.I: 1.31, 5.95) when compared to women who did not have a pregnancy occurring during the follow-up time. Longer time on ART before LTFU, higher baseline CD4, initiating ART after 2015, and being on a longer refill schedule (a proxy for stable patients) were associated with a higher hazard of ART resumption following LTFU. The hazard of ART resumption was lower the longer a patient had been LTFU (Table 3).

## Discussion

Using linked demographic surveillance and health facility data, we investigated patterns of HIV testing and engagement in care for pregnant and breastfeeding women. Our findings showed a reduction in the number of women testing positive each year during the MTCT risk period. We also identified four patterns of engagement in care during the MTCT risk period representing varying times of seroconversion and different levels of care stability following ART initiation, with women’s age, marital status, baseline CD4 and the year of delivery predicting these patterns. We also found that following discontinuation of HIV care, ART resumption was more likely for women who had a new pregnancy.

An estimated 15% of pregnancies resulting in a live birth recorded in the Agincourt HDSS were to women who had utilised HIV-related healthcare. Of the pregnancies linked to HIV-related healthcare utilisation, 51% were to previously diagnosed women and 49% had no previous HIV-related healthcare data and were presumed to be new diagnoses. Although some of the women who were previously diagnosed had disengaged from care, encouragingly most resumed treatment over the study period.

Reengagement in care is becoming a more common phenomenon as more healthy patients initiate ART [15]. Our finding that a new pregnancy was associated with an increased probability of resumption was probably due to increased interaction with the health system, as well as a desire to protect their baby. Whilst about half of these women resumed ART before delivery, research shows that ART initiation *early* in the pregnancy is most effective at reducing MTCT risk [14, 39]. Furthermore, whilst ART resumption is a positive outcome, it represents a programmatic failure of Option B+ which aims to keep women on ART for life and extend protection throughout subsequent pregnancies. Treatment interruptions may also contribute to drug resistance [40, 41]. We found that 66.1% of WLHIV who were LTFU when they became pregnant and 51.5% of WLHIV but not on ART when they became pregnant were still not on treatment by delivery, indicating that ART programmes still have much work to do to initiate and keep less compliant patients on ART.

The cumulative probability of a new HIV diagnosis occurring during the MTCT risk period was estimated as 5.9% during pregnancy, increasing to 7.5% by the breastfeeding cessation. The proportion of women testing positive for HIV during pregnancy increased until 2016, reaching 7%, and then declined in 2017 to 5%. These are likely to be underestimates as some women might have attended private facilities or facilities outside the HDSS. Additionally, while UNAIDS estimated that 90.1% of pregnant women in South Africa were tested for HIV in 2019 [1], without a comprehensive HIV testing database where both negative and positive results are recorded, it is likely that not all seroconversions were captured. Nonetheless, the reduction in the number of women testing HIV positive in 2017 is encouraging. Option B+ and treat all were implemented as part of the paradigm shift towards using treatment as prevention. South Africa national guidelines moved to Option B+ in 2015 and to universal treatment in 2016 [6, 42], all of which have been widely adopted in the area covered by the Agincourt HDSS. Other countries have reported a surge in the volume of pregnant women testing positive and utilising PMTCT services as more women became eligible for ART following the implementation of Option B+ [43]. This was followed by a steady decline as more WLHIV became aware of their HIV status [44]. Our data suggests, there was a further reduction in 2018, however, further research and monitoring of this trend will be needed.

Over half of HIV-exposed pregnancies occurred to women who already knew their HIV status. There is a general recognition that the number of HIV-positive women having children who already know their HIV-status is increasing [44, 45], illustrating the importance of keeping women engaged in care and virally suppressed. Our finding that older age was associated with sustained retention on ART aligns with other studies [46–49]. Later year of delivery was also found to be associated with early stable ART with the odds of late ART decreasing over time potentially showing the benefits of simplified treatment guidelines under Option B+. We found that married women were more likely to initiate ART late which other studies have reported citing the need for their partner’s support [50–52]. As such, interventions that improve partner support such as couple testing or joint counselling are important to encourage early ART initiation for married women [10, 53, 54].

Teenage mothers were more likely to have a first positive test recorded postnatally which might suggest late seroconversion, late testing, or poorer health seeking behaviour, all of which have been reported as important factors among this age group in similar settings [55–57]. Older women were more likely to have already seroconverted or to be on treatment at the beginning of their pregnancy which possibly reflects their longer time at risk for HIV. There were lower odds of postnatal seroconversion in later years, possibly indicating reduced incidence of HIV in the wider population.

This study has some limitations. Firstly, the health facilities used may not capture every sentinel event as a small proportion of HDSS residents receive ART through private facilities or public facilities outside the HDSS. Furthermore, we did not have treatment adherence data. The assumption that a patient was in care until their next scheduled visit might therefore misclassify some patients. During the linkage process a very small proportion of residents declined to be linked and we did not manage to link everyone that claimed to be an HDSS resident. These linkage errors might bias these results downwards. Finally, there are biases arising from using routinely collected data. If women testing positive did not self-report a previous diagnosis, we would have erroneously categorised them as newly diagnosed. Additionally, if a woman tested positive but did not report to the PMTCT clinic to start treatment then she would have been missed as this data would only be available in the testing registers which were not linked to HDSS data. Finally, data from 2018 does not include clinic data past July 2018 or pregnancy data past November 2018, as such HIV incidence reduction seen in our analysis in 2018 might be an effect of the data.

This study also has some strengths. To our knowledge, this is the first study to use linkage of population and clinical records to estimate HIV incidence in pregnant women in SSA. The method of linking routinely collected data to answer this question shows promise and should be considered in future research. The use of multiple data sources also means we were able to crosscheck matching data and had access to new pregnancies data and socioeconomic variables which are not always available in clinical data.

## Conclusions

In conclusion, our study shows the importance of new pregnancies in encouraging treatment resumption for women previously LTFU. This coupled with the reduction in the number and proportion of pregnant and postpartum women testing HIV positive should be considered as successes of the Option B+ programme. Our study also shows the growing scale of reengagement in care. Treatment programmes will need interventions to keep pregnant women on ART after delivery and ensure that pregnant women previously in care are reengaged promptly for their own health and to protect their unborn infants.

## Supplementary Information

**Additional file 1.** Flowchart showing data sources and data used for all analyses. A flowchart that illustrates the different databases and data that were used to conduct the different analyses for this manuscript.

**Additional file 2.** Kaplan-Meier curves showing the probability of ART initiation following an HIV-positive test during pregnancy stratified by timing of the positive test and year of delivery. A panel of graphs showing ART initiation stratified by trimester at receipt of a positive HIV result and year of delivery.

**Additional file 3.** Chronograms of engagement in care during the vertical risk transmission period by each engagement cluster. A panel of graphs showing engagement cluster chronograms.

**Additional file 4.** Histogram of number of resumptions following a new pregnancy stratified by type of resumption (reengagement vs transfer) and year of delivery. A panel of graphs showing the frequency of clinic transfer or reengagement in care stratified by the year of delivery.

**Additional file 5.** Factors associated with membership in each identified engagement cluster. A table showing factors associated with membership in each engagement cluster.

## Data Availability

The data that support the findings of this study are available from MRC/Wits Rural Public Health and Health Transitions Research Unit (Agincourt), but restrictions apply to the availability of these data, which were used under license for the current study, and so are not publicly available. Data are however available by request here: https://www.agincourt.co.za/?page_id=1883 and with permission of the MRC/Wits Rural Public Health and Health Transitions Research Unit (Agincourt).
