# Supplementary figures and images for: Patterns of engagement in HIV care during pregnancy and breastfeeding: findings from a cohort study in North-Eastern South Africa
Source: BMC Public Health. 2021 Sep 21;21:1710. doi: 10.1186/s12889-021-11742-4 (PMC8454048; doi:10.1186/s12889-021-11742-4)

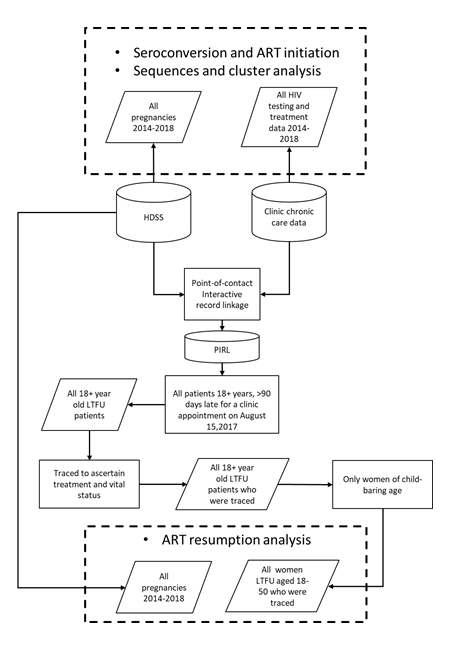

Supplement: Supplementary file 1 — Additional file 1. Flowchart showing data sources and data used for all analyses. A flowchart that illustrates the different databases and data that were used to conduct the different analyses for this manuscript. [file 12889_2021_11742_MOESM1_ESM.png]

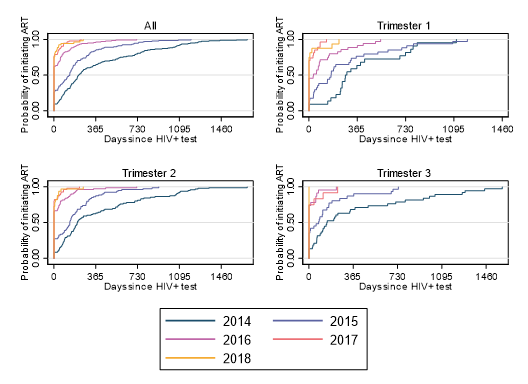

Supplement: Supplementary file 2 — Additional file 2. Kaplan-Meier curves showing the probability of ART initiation following an HIV-positive test during pregnancy stratified by timing of the positive test and year of delivery. A panel of graphs showing ART initiation stratified by trimester at receipt of a positive HIV result and year of delivery. [file 12889_2021_11742_MOESM2_ESM.png]

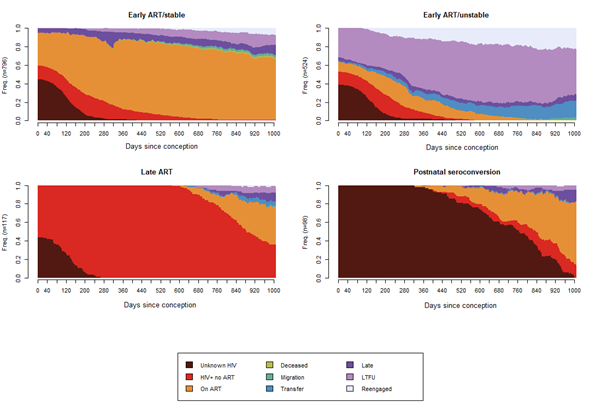

Supplement: Supplementary file 3 — Additional file 3. Chronograms of engagement in care during the vertical risk transmission period by each engagement cluster. A panel of graphs showing engagement cluster chronograms. [file 12889_2021_11742_MOESM3_ESM.png]

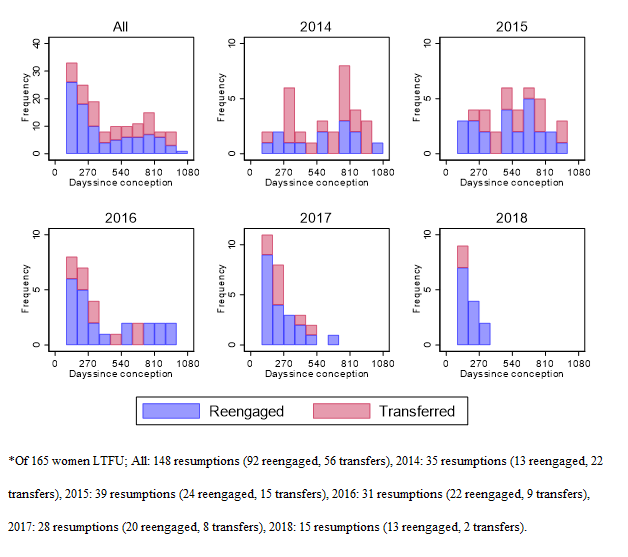

Supplement: Supplementary file 4 — Additional file 4. Histogram of number of resumptions following a new pregnancy stratified by type of resumption (reengagement vs transfer) and year of delivery. A panel of graphs showing the frequency of clinic transfer or reengagement in care stratified by the year of delivery. [file 12889_2021_11742_MOESM4_ESM.png]
