# Supplementary material for: Patterns of engagement in HIV care during pregnancy and breastfeeding: findings from a cohort study in North-Eastern South Africa
Source: BMC Public Health. 2021 Sep 21;21:1710. doi: 10.1186/s12889-021-11742-4 (PMC8454048; doi:10.1186/s12889-021-11742-4)
Supplement: Supplementary file 5 — Additional file 5. Factors associated with membership in each identified engagement cluster. A table showing factors associated with membership in each engagement cluster. [file 12889_2021_11742_MOESM5_ESM.docx]

**Additional File 4:** Factors associated with membership in each identified engagement cluster.

|  |  |  | Clusters |  |  |  |  |  |
| --- | --- | --- | --- | --- | --- | --- | --- | --- |
|  | **Early ART /stable** | **p-value** | **Early ART /unstable** | **p-value** | **Late ART** | **p-value** | **Postnatal HIV** | **p-value** |
|  | N=796 |  | N=524 |  | N=117 |  | N=98 |  |
|  | **aOR** |  | **aOR** |  | **aOR** |  | **aOR** |  |
| Age |  |  |  |  |  |  |  |  |
| 15-19 | Reference | __ | Reference | __ | Reference | __ | Reference | __ |
| 20-24 | 1.23 | 0.4 | 1.11 | 0.7 | 1.18 | 0.7 | 0.45 | 0.04 |
| 25-29 | 1.69 | 0.054 | 1.07 | 0.8 | 0.6 | 0.3 | 0.3 | 0.002 |
| 30-34 | 2.08 | 0.008 | 0.8 | 0.4 | 1.04 | >0.9 | 0.23 | <0.001 |
| 35-39 | 2.1 | 0.009 | 0.9 | 0.7 | 0.97 | >0.9 | 0.09 | <0.001 |
| 40+ | 2.71 | 0.003 | 0.71 | 0.3 | 0.52 | 0.3 | 0.22 | 0.011 |
| Marital status |  |  |  |  |  |  |  |  |
| Single | Reference | __ | Reference | __ | Reference | __ | Reference | __ |
| Married | 1.05 | 0.8 | 0.71 | 0.086 | 1.98 | 0.032 | 1.22 | 0.6 |
| Informal union | 0.99 | >0.9 | 0.94 | 0.7 | 1.25 | 0.4 | 1.01 | >0.9 |
| Separated | 0.91 | 0.7 | 1.34 | 0.2 | 0.62 | 0.3 | 0.58 | 0.4 |
| Divorced | 1.03 | >0.9 | 0.99 | >0.9 | 0.92 | 0.9 | 1.16 | 0.8 |
| Widowed | 0.95 | 0.9 | 0.8 | 0.5 | 2.15 | 0.1 | 0.9 | 0.9 |
| Delivery year |  |  |  |  |  |  |  |  |
| 2014 | Reference | __ | Reference | __ | Reference | __ | Reference | __ |
| 2015 | 1.84 | <0.001 | 1.04 | 0.8 | 0.34 | <0.001 | 0.7 | 0.2 |
| 2016 | 2.23 | <0.001 | 1.35 | 0.06 | 0.05 | <0.001 | 0.42 | 0.005 |
| 2017 | 2.71 | <0.001 | 1.21 | 0.3 | 0 | __ | 0.33 | 0.002 |
| 2018 | 2.97 | <0.001 | 1.33 | 0.1 | 0 | __ | 0 | __ |
| Baseline CD4 |  |  |  |  |  |  |  |  |
| <100 | Reference | __ | Reference | __ | Reference | __ | Reference | __ |
| 100-200 | 1.06 | 0.8 | 1.11 | 0.6 | 1.06 | 0.9 | 0.37 | 0.036 |
| 201-350 | 1.18 | 0.4 | 0.88 | 0.5 | 1.49 | 0.4 | 0.52 | 0.091 |
| 351-500 | 1.14 | 0.5 | 0.81 | 0.3 | 1.49 | 0.4 | 0.79 | 0.6 |
| >500 | 0.89 | 0.6 | 0.83 | 0.4 | 2.62 | 0.031 | 0.91 | 0.8 |
